# Lectin activity of the pneumococcal pilin proteins

**DOI:** 10.1038/s41598-017-17850-9

**Published:** 2017-12-19

**Authors:** Christopher J. Day, Adrienne W. Paton, Richard M. Harvey, Lauren E. Hartley-Tassell, Kate L. Seib, Joe Tiralongo, Nicolai Bovin, Silvana Savino, Vega Masignani, James C. Paton, Michael P. Jennings

**Affiliations:** 1 Institute for Glycomics, Griffith University, Gold Coast, QLD 4222 Australia; 2 Research Centre for Infectious Diseases, Department of Molecular and Cellular Biology, University of Adelaide, Adelaide, 5005 Australia; 3 Shemyakin Institute of Bioorganic Chemistry, Russian Academy of Sciences, Moscow, Russia; 4 GSK Vaccines, Via Fiorentina 1, Siena, Italy

## Abstract

*Streptococcus pneumoniae* is a leading cause of morbidity and mortality globally. The Pilus-1 proteins, RrgA, RrgB and RrgC of *S. pneumoniae* have been previously assessed for their role in infection, invasive disease and as possible vaccine candidates. In this study we have investigated the glycan binding repertoire of all three Pilus-1 proteins, identifying that the tip adhesin RrgA has the broadest glycan recognition of the three proteins, binding to maltose/cellobiose, α/β linked galactose and blood group A and H antigens. RrgB only bound mannose, while RrgC bound a subset of glycans also recognized by RrgA. Adherence of *S. pneumoniae* TIGR4 to epithelial cells was tested using four of the oligosaccharides identified through the glycan array analysis as competitive inhibitors. The blood group H trisaccharide provided the best blocking of *S. pneumoniae* TIGR4 adherence. Adherence is the first step in disease, and host glycoconjugates are a common target for many adhesins. This study has identified Pilus-1 proteins as new lectins involved in the targeting of host glycosylation by *S. pneumoniae*.

## Introduction

*Streptococcus pneumoniae* is one of the leading causes of morbidity and mortality worldwide^1–3^. *S. pneumoniae* causes a range of diseases including pneumonia, meningitis, septicemia and otitis media, and produces a range of virulence factors including the toxin pneumolysin, pneumococcal surface protein A and pilus^2,4–6^. The current *S. pneumoniae* vaccines target the capsular polysaccharide, but cover only a subset of the 97 known capsular serotypes. Differences in serotype distribution between developed and developing countries, and serotype replacement in response to widespread use of the vaccines, are reducing the overall impact of the vaccines on the burden of pneumococcal disease^1,3^.

Expression of the pneumococcal Pilus-1, composed of three proteins RrgA, RrgB and RrgC, has been linked to pneumococcal meningitis in mouse infection models^5–7^. Pilus-1 was found to be required for the bacteria to breach the blood brain barrier^5^. The Pilus-1 protein complex consists of RrgB as the shaft protein, with RrgA as the tip adhesin and RrgC, which serves as a pilus anchor at the cell surface^6–8^. The *S. pneumoniae* Pilus-1 protein complex has been proposed as a novel vaccine target^9,10^. The RrgA and RrgB proteins were found to produce cross-protecting antibodies that led to blocking of adherence of *S. pneumoniae* to cells in culture and resulted in the opsonophagocytosis of *S. pneumoniae* ^9,11^. RrgA is also involved in regulation of the host immune response to *S. pneumoniae* by binding to both MAC-1 (complement receptor 3, CD11b/CD18)^12^ and Toll-like receptor 2^13^. RrgA has also been shown to interact directly with cultured epithelial cells and extracellular matrix components including fibronectin and collagen^10^.

All of the known proteins that interact with RrgA are glycoproteins, indicating a potential role of the oligosaccharides in the interactions. In this study we aim to identify glycan targets of the Pilus-1 protein complex of *S. pneumoniae*.

## Results

### RrgA, RrgB and RrgC proteins bind glycans

Glycan array analysis of the three Pilus-1 proteins from *S. pneumoniae* TIGR4 and RrgA from strain SPEC6B revealed differential glycan recognition between the four proteins tested. *S. pneumoniae* TIGR4 RrgB bound the least number of glycans, with only an α-mannobiose recognized (Table 1 and Dataset S1). RrgA from *S. pneumoniae* TIGR4 and RrgA from *S. pneumoniae* SPEC6B bound to a set of overlapping glycans including terminal galactose structures with both α and β linkages, glucose/maltose-related structures and blood group A (7 K and 392) and blood group H(O) (7 A) antigens (Table 1 and Dataset S1). No interactions with terminal galactose structures were observed when galactose was directly linked to glucose. *S. pneumoniae* SPEC6B RrgA also had binding to β-linked mannose and β-linked N-acetylglucosamine structures not bound by the TIGR4 RrgA. RrgC bound to structures that are the same or very similar to RrgA from TIGR4 with additional recognition of blood group B antigen (483), Lewis B (496) and hyaluronic acid (14I) (Table 1 and Dataset S1).

**Table 1:** Glycans bound by Rrg proteins in glycan array analysis.

No.	Structure	RrgA T4	RrgA SPEC6B	RrgB T4	RrgC T4
**MONOSACCHARIDES**					
3	Galβ-sp3	1587 ± 308	369 ± 188	108 ± 88.9	2165 ± 450
14	Glc*N*(Gc)β-sp4	157 ± 171	2112 ± 110	−37.5 ± 63.5	191.3 ± 124
18	Manβ-sp4	166.3 ± 198	2532 ± 436	−113.5 ± 68.8	600.5 ± 650
22	Glc*N*Acβ-sp4	1140 ± 302	55.5 ± 413	227 ± 190	57.5 ± 184
**Terminal Galactose**					
76	Galα1-3Galβ-sp3	1918.3 ± 154	142 ± 120	−67.3 ± 347	284.5 ± 180
80	Galα1-3Glc*N*Acβ-sp3	90.75 ± 196	270.5 ± 379	40.5 ± 388	1627 ± 122
88	Galβ1-3Gal*N*Acβ-sp3	1013 ± 101	1251 ± 87.0	15.3 ± 204	1402 ± 204
89	Galβ1-3Gal*N*Acα-sp3	1098 ± 277	2495 ± 322	−7 ± 266	1033 ± 295
94	Galβ1-4Galβ-sp4	1218 ± 458	186 ± 248	73.5 ± 247	374.3 ± 190
100	Galβ1-6Galβ-sp4	2197 ± 486	1027 ± 149	144.8 ± 114	3443.5 ± 523
220	Galα1-3Galβ1-4Glcβ-sp2	142.9 ± 201	151.3 ± 152	63.75 ± 95.5	1283 ± 310
222	Galα1-3Galβ1-4Glc*N*Acβ-sp3	99.0 ± 237	150.5 ± 162	−107.8 ± 305	3108 ± 641
373	Galα1-3Galβ1-4Glc*N*Acβ1-3Galβ-sp3	1802.5 ± 276	1270 ± 194	62.3 ± 148	2374 ± 839
381	Galβ1-3Glc*N*Acβ1-6Galβ1-4Glc*N*Acβ-sp2	1985.4 ± 280	2600 ± 232	−72.3 ± 166	567.0 ± 259
383	Galβ1-4Glc*N*Acβ1-3Galβ1-4Glcβ-sp2	126.0 ± 279	−510.3 ± 557	157 ± 414	1799 ± 160
489	Galβ1-4Glc*N*Acβ1-3(Glc*N*Acβ1-6)Galβ1-4Glc*N*Ac-sp2	78.25 ± 96.3	437.3 ± 435	188 ± 122	1107 ± 142
501	Galβ1-3Gal*N*Acβ1-3Galα1-4Galβ1-4Glcβ-sp4	116.0 ± 166	1012 ± 77.5	57.3 ± 185	187.0 ± 155
504	(A-GN-M)_2_-3,6-M-GN-GNβ-sp4	197.8 ± 201	105.3 ± 76.31241	74.5 ± 78.5	1906 ± 180
1 G	Galβ1-3Glc*N*Acβ1-3Galβ1-4Glc	4605 ± 171	1613 ± 150	14 ± 165	606.1 ± 738
2 G	Galβ1-3Glc*N*Acβ1-3Galβ1-4Glc*N*Acβ1-6(Galβ1-3Glc*N*Acβ1-3)Galβ1-4Glc	1344 ± 583	2655 ± 197	−127 ± 128	301.3 ± 300
**Terminal N-Acetylgalactosamine**					
2 F	Gal*N*Acα1-3Galβ1-4Glc	1221.5 ± 204	2988 ± 200	144.3 ± 246	595.6 ± 614
**Fucosylated**					
392	Fucα1-2(Gal*N*Acα1-3)Galβ1-3Gal*N*Acα-sp3	1366 ± 296	2274 ± 208	140.6 ± 165	2350 ± 329
480	Fucα1-2Galβ1-3Glc*N*Acβ1-3Galβ1-4Glc*N*Acβ-sp2	315.25 ± 272.0421	122.5 ± 399	264 ± 427	21.5 ± 227
483	Fucα1-3(Fucα1-2 (Galα1-3)Galβ1-4)Glc*N*Acβ-sp3	327.3 ± 438	−17.5 ± 311	170.5 ± 230	1178 ± 258
496	Fucα1-2Galβ1-3(Fucα1-4)Glc*N*Acβ1-3Galβ1-4Glcβ-sp4	274.5 ± 331	286 ± 105	38.5 ± 75.5	1192 ± 322
538	Le^x^1-6′(Le^c^1-3′)Lac-sp4	1261.5 ± 346	118.3 ± 206	93.6 ± 114	131.3 ± 204
539	LacNAc1-6′(Le^d^1-3′)Lac-sp4	206.8 ± 210	1393 ± 160	59.3 ± 247	−49.0 ± 602
7A	Fucα1-2Galβ1-3Glc*N*Acβ1-3Galβ1-4Glc	1897.3 ± 295	2619 ± 95.1	−41.8 ± 91.7	130.3 ± 283
7K	Gal*N*Acα1-3(Fucα1-2)Gal	1159.0 ± 162	2302 ± 149	69.8 ± 176	472.8 ± 587
8A	SO_3_-3Galβ1-3(Fucα1-4)Glc*N*Ac	918.5 ± 84.6	1638 ± 272	101.5 ± 142	29.0 ± 112
8J	Fucα1-2Galβ1-4(Fucα1-3)Glc*N*Acβ1-3(Fucα1-2)Galβ1-4Glc	1692.3 ± 305	138 ± 338	−120.8 ± 125	245.3 ± 265
8K	Galβ1-4(Fucα1-3)Glc*N*Acβ1-6(Galβ1-4Glc*N*Acβ1-3)Galβ1-4Glc	170.0 ± 177	7622 ± 1317	2 ± 141	807.5 ± 791
**Mannose**					
120	Manα1-3Manβ-sp4	18.0 ± 406	90 ± 222	1332 ± 275	41.1 ± 54
**Terminal N-Acetylglucosamine**					
117	Glc*N*Acβ1-4Glc*N*Acβ-sp4	145.6 ± 238	5714 ± 1240	−73 ± 113	226.5 ± 179
118	Glc*N*Acβ1-6Gal*N*Acα-sp3	1322.5 ± 336	−61 ± 175	−56.3 ± 99.6	122.3 ± 31
253	Glc*N*Acβ1-6Galβ1-4Glc*N*Acβ-sp2	54.75 ± 212	25.8 ± 60.1	−160 ± 74.9	1641 ± 117
505	(GN-M)_2_-3,6-M-GN-GNβ-sp4	244.5 ± 183	2695 ± 351	28.0 ± 214	106.3 ± 180
**Glucose**					
112	Glcβ1-6Glcβ-sp4	1284.8 ± 445	1863 ± 44.6	−154.5 ± 176	506.5 ± 418
390	(Glcα1-4)_4_β-sp4	930.5 ± 94.7	1700 ± 311	89.3 ± 413	187.3 ± 154
**High molecular weight Carageenan and Glycoaminoglycans (GAGS)**					
14I	HA 1600000 da 2.5 mg/ml	985 ± 884	187.8 ± 164	422.5 ± 698	1270 ± 147

Values are global background subtracted. Red indicates binding. Binding is determined by positive interaction in three replicate array experiments. Positive interactions are determined by a background subtracted fluorescence value significantly above background subtracted fluorescence of negative control spots (average background fluorescence from 20 spots + 3 standard deviations).

### Surface Plasmon Resonance analysis of RrgA and glycans identified by array analysis

To validate the glycan array results and to determine the dissociation equilibrium constant (K_D_) of the interactions, surface plasmon resonance was performed between free oligosaccharides and captured RrgA proteins (Table 2). The highest affinity (smallest K_D_) interaction was between RrgA proteins from both *S. pneumoniae* TIGR4 and SPEC6B and the H trisaccharide type 3/4 (Table 2).

**Table 2:** Surface plasmon resonance results for RrgA proteins and glycans^a^.

Oligosaccharide	RrgA TIGR4	RrgA SPEC6B
Blood group A tri	916.5 nM ± 66.4	1.4 μM ± 0.29
Blood group A type3/4	870.7 nM ± 286	643.6 nM ± 157
Blood group H type 3/4	89.4 nM ± 16	358.1 nM ± 51
Lewis X	434.0 nM ± 118	363.3 nM ± 107
Lacto-N-tetraose	1.03 μM ± 0.05	868.6 nM ± 454
maltobiose	1.16 μM ± 0.09	964.0 nM ± 226
cellobiose	1.48 μM ± 251	582.8 nM ± 155
chitobiose	409.3 nM ± 160	426.2 nM ± 144
lactose	NCDI	NCDI

^a^Data are dissociation equilibrium constants (K_D_) of the interactions between free oligosaccharides and captured RrgA proteins; NCDI: no concentration-dependent interaction detected.

### Inhibition of adherence of RrgA-expressing *S. pneumoniae* TIGR4 using free oligosaccharides

*S. pneumoniae* TIGR4 and *S. pneumoniae* TIGR4Δ*rrgA* were tested for adherence differences using A549 human lung carcinoma and Detroit 562 pharyngeal carcinoma cell lines. These cell lines are representative of the two most important sites for colonization/infection of humans by *S. pneumoniae*. *S. pneumoniae* TIGR4Δ*rrgA* was significantly less adherent to both cell lines, with RrgA contributing to around 50% of the adherence of *S. pneumoniae* TIGR4 to A549 cells and 70% of the adherence to Detroit 562 cells (Table 3).

**Table 3:** Adherence of TIGR4 vs TIGR4Δ*rrgA*.

	A549 adherence (% TIGR4 ± SEM)^*a*^	Detroit 562 (% TIGR4 ± SEM)^*b*^
TIGR4	100 ± 5.4	100 ± 19.8
TIGR4Δ*rrgA*	52.7 ± 3.9	27.1 ± 7.4
Significance	*P* < 0.001	*P* < 0.01

Data are the average of two separate experiments consisting of 4 individual wells (experiment 1) and 5 individual wells (experiment 2) (n = 9). ^a^100% = 1.97 × 10^7^ CFU per well. ^b^100% = 8.70 × 10^6^ CFU per well.

The Detroit 562 and A549 cell lines were examined for the presence of RrgA target glycans using lectin array analysis (see Dataset S2). Lectins recognizing terminal β-linked galactose were observed for both cells. The A549 cells were recognized by the lectins AAA and RSL, both of which recognize structures containing α1-2 linked fucose, while the Detroit 562 cells did not (Dataset S2). Lectins with α1-2 linked fucose specificity recognize blood group antigens, particularly the H (O) blood group antigens.

Adherence of *S. pneumoniae* TIGR4 to A549 cells was then re-examined using four of the oligosaccharides identified through the glycan array analysis as competitive inhibitors. The blood group H type 3/4 trisaccharide provided the best blocking of *S. pneumoniae* TIGR4 adherence, with 67.7% inhibition (Table 4; *P* < 0.001). In addition, maltose, cellobiose and blood group A tetrasaccharide all reduced adherence by around 56% (Table 4; *P* < 0.001 in all cases). Interestingly, the residual A549 adherence observed for TIGR4Δ*rrgA* (approximately 50% relative to wild type TIGR4) could also be competitively inhibited by a further 30–46% by cellobiose and blood group A tetrasaccharide and H trisaccharide (Table 4; *P* < 0.001).

**Table 4:** Effect of glycans on adherence of TIGR4 to A549 cells.

Strain/glycan	A549 adherence (% control ± SEM)^*a*^	Significance vs untreated
TIGR4^*a*^	100 ± 9.2	—
TIGR4 + 200 μM lactose	85.5 ± 11.0	Not significant
TIGR4 + 200 μM maltose	43.7 ± 9.2	*P* < 0.001
TIGR4 + 200 μM cellobiose	44.6 ± 3.1	*P* < 0.001
TIGR4 + 200 μM BGA type 3/4	44.0 ± 2.5	*P* < 0.001
TIGR4 + 200 μM BGH type3/4	32.3 ± 2.7	*P* < 0.001
TIGR4Δ*rrgA* ^*b*^	100 ± 3.9	—
TIGR4Δ*rrgA* + 200 μM cellobiose	62.4 ± 4.2	*P* < 0.001
TIGR4Δ*rrgA* + 200 μM BGA type 3/4	69.6 ± 2.1	*P* < 0.001
TIGR4Δ*rrgA* + 200 μM BGH type3/4	53.5 ± 1.6	*P* < 0.001

Data are the average from four individual wells performed as replicates (n = 4). ^*a*^100% = 3.50 × 10^7^ CFU per well. ^*b*^100% = 1.97 × 10^7^ CFU per well. BGA: Blood group A, BGH: Blood group H.

## Discussion

Adherence to host tissues is the first step in disease and host glycoconjugates are a common target for numerous bacterial adhesins^14–16^, including pili/fimbriae^17,18^. Pilus-1 of *S. pneumoniae* has been shown to interact with Toll-like receptor 2 and MAC-1^12,13^ and epithelial cells and ECM components^10^, all of which are glycosylated.

The glycan array analysis of RrgA from *S. pneumoniae* TIGR4 and SPEC6B identified a cluster of oligosaccharide binding that was consistent between the two proteins. These proteins have previously been shown to bind to both cells and ECM components equally, indicating recognition of uncapped β-linked galactose. Binding of *S. pneumoniae* RrgA to β-linked galactose and blood group A glycan is consistent with interactions between *S. pneumoniae* and a wide variety of cell surfaces in the human host.

The array binding was investigated further using surface plasmon resonance analysis. Binding to blood group antigens, β-galactose, maltose, cellobiose and GlcNAc were confirmed through SPR analysis, with the highest affinity interaction observed occurring between RrgA from TIGR4 with the H-type 3/4 oligosaccharide (Table 2). As observed in the array experiment, no concentration dependent interaction was observed using SPR for terminal galactose with underlying glucose (lactose; Table 2). The SPR analysis found high affinity binding between RrgA and Lewis X (K_D_ < 500 nM; Table 2).

The significance of these interactions in terms of interaction with host surfaces was further confirmed by competitive inhibition of TIGR4 adherence to A549 cells by exogenous maltose, cellobiose, blood group A type 3/4 and blood group H type 3/4 oligosaccharides. Interestingly, although RrgA is thought to be the most important component of the Pilus-1 structure in terms of mediating adherence, the residual A549 adherence exhibited by TIGR4Δ*rrgA* could be inhibited further, albeit to a slightly lesser extent, by the three exogenous oligosaccharides that were most effective against TIGR4. There are multiple *S. pneumoniae* surface proteins that have been shown to have a role in adherence to host epithelial cells, most notably CbpA, which is known to bind to cell surface glycoconjugates^19^, indicating that the residual binding observed in the TIGR4Δ*rrgA* is likely to be another adhesin.

The direct interaction between RrgA and Lewis X was not seen on the array, but Lewis X was a terminal group on several of the larger fucosylated structures identified (Table 1; 8 J and 8 K). This may indicate that the binding of Lewis X requires a specific presentation that is available in solution, but not available on the array except as a part of a larger structure. Lewis X is a member of the Lewis system histo-blood group antigens, which were originally identified on RBCs where they are acquired from the plasma as glycolipids^20^ and is expressed on most cell types but is over-expressed by human tumor cells from various sites^21^.

*S. pneumoniae* is a host-adapted pathogen that infects humans so it is unexpected to observe the binding of the Rrg proteins to many non-human and non-mammalian glycans. Rrg proteins recognised maltose and cellulose saccharides, repeating units of glucose typically produced by plants, algae and some bacteria^22^. This interaction may allow *S. pneumoniae* to interact with other bacterial species either directly or to the matrix of bacteria in biofilm communities.

RrgC and RrgA also bound to the non-human terminal α1-3-linked galactose structures. RrgC bound four of the 11 terminal α1-3-linked galactose structures present on the array, while RrgA only bound 1-2 terminal α1-3-linked galactose structures on the array (Table 1). This binding does not correlate with the host adapted nature of *S. pneumoniae* with α1-3-linked galactose widely expressed in non-human mammals but not in humans. The expression of α1-3-linked galactose is only observed in humans that produce blood group B antigens, structures not recognised on the array by the Rrg proteins.

The Pilus-1 complex of *S. pneumoniae* is known to be an important mediator of adherence of *S. pneumoniae* to host epithelial cells. This binding appears to be glycan related, as RrgA was found to have lectin activity, recognising a range of common host cell surface glycans. All three proteins that make up the Pilus-1 complex recognise glycans as recombinant soluble proteins. Thus, we have identified three new lectins produced by *S. pneumoniae*, with the pilius tip protein RrgA directly involved in interactions between pathogen and host.

## Materials and Methods

### Cloning, expression and purification of Rrg proteins

Genes were cloned into pET protein expression vectors and proteins were expressed and purified as previously described^10^.

### Glycan Array

Glycan array slides were printed using SuperEpoxy 3 activated substrates as previously described^23^ using an ArrayIt Spotbot Extreme 3 contact printer with solid metal pins. The Glycan array binding experiments were performed and analysed as previously described^16^. Briefly 1 μg of protein in 1xPBS containing 1 mM MgCl_2_ and 1 mM CaCl_2_ was pre-complexed with mouse anti-His antibody (Cell signaling), Alexa555 rabbit anti-mouse IgG and Alexa555 goat anti-rabbit IgG and allowed to bind to a pre-blocked (1% bovine serum albumin in PBS) for 15 minutes. Slides were washed three times for 2 minutes in 1xPBS, dried by centrifugation and scanned and analysed using the Scan Array Express software package (Perkin Elmer) and Microsoft Excel for statistical analysis (Student’s unpaired *t-*test of fluorescence of background spots vs fluorescence of glycan printed spots).

### Surface Plasmon Resonance analysis

The interactions between the Rrg proteins and test glycans were analysed using surface plasmon resonance (SPR) using a Biacore T100 system as described Shewell *et al*.^16^ with the following modifications. Proteins were immobilised onto a CM5 chip at pH 4.5, flow rate of 10 µL/min for 420 seconds with an ethanolamine blank flow cell as a control. Glycans were tested between 160 nM and 100 µM. All data was double reference subtracted.

### Mutagenesis of *rrgA* in *S. pneumoniae* TIGR4

The *rrgA* gene in TIGR4 was deleted in-frame and replaced with an erythromycin resistance cassette (*erm*) by direct transformation with a linear DNA fragment constructed by overlap-extension PCR, essentially as previously described^24^. This involved using primer pairs rrgAF/rrgAermR and rrgAermF/rrgAR to amplify 5′ and 3′ regions flanking *rrgA* from TIGR4 template DNA, and J214/J215 to amplify *erm* from pVA838 (Table 5). Primers included overlapping sequences such that the three PCR products could be fused in a second round of PCR using primer pair rrgAF/rrgAR Erythromycin-resistant transformants were screened by PCR for deletion of *rrgA*, confirmed by DNA sequencing and designated TIGR4Δ*rrgA*.

**Table 5:** Oligonucleotide primers.

Primer	Sequence 5′-3′
rrgAF	CAGAAACTAGAGAGCAGAAGTG
rrgAR	CTGACGGCTAGTTAGTAATGC
rrgAermR	TTGTTCATGTAATCACTCCTTCTATTCATAGAACAAGTTAATCCTTC
rrgAermF	CGGGAGGAAATAATTCTATGAGAGTGTAGAAATGATATCTATGTTC
J214	GAAGGAGTGATTACATGAACAA
J215	CTCATAGAATTATTTCCTCCCG

### Competitive inhibition of adherence of *S. pneumoniae* with free glycans

Adherence assays on A549 (human type II pneumocyte) and Detroit 562 (human nasopharyngeal carcinoma) cells were carried out as previously described^25^. A549 and Detroit 562 cells were grown in Dulbecco’s Modified Eagle Medium (DMEM; Gibco), or a 1:1 mix of DMEM and Ham’s F-12 Nutrient Mixture (Gibco), respectively, supplemented in both cases with 5% fetal bovine serum, 2 mM L-glutamine, 50 IU of penicillin and 50 μg/ml streptomycin. Confluent monolayers in 24-well plates were washed with PBS and infected with pneumococci (approximately 5 × 10^5^ CFU per well) in a 1:1 mixture of the respective culture medium (without antibiotics) and C + Y medium, pH 7.4^23^. Plates were centrifuged at 500 × *g* for 5 min, and then incubated at 37 °C in 5% CO_2_ for 2.5 h. Monolayers were washed 3 times in PBS, and adherent bacteria were released by treatment with 100 μl trypsin/EDTA, followed by 400 μl 0.025% Triton X-100. Lysates were serially diluted and plated on blood agar to enumerate adherent bacteria. Total adherence (mean ± SEM for 4-9 replicates) was expressed as a percentage of that for TIGR4 (or TIGR4Δ*rrgA* where appropriate) without additives. For inhibition assays, free oligosaccharides were used at a final concentration of 200 μM (>100 × K_D_) throughout the adherence assay. Data were analysed using Student’s un-paired *t*-test (two-tailed).

### Lectin array analysis of Detroit 562 and A549 cells

The two cells lines used for adherence assays, A549 and Detroit 562, were analysed for cell surface glycans using lectin arrays. Lectin arrays were printed using an ArrayJet Argus Marathon Inkjet Bio-Printing System on Arrayit SME3 substrates. The lectins are immobilized to the epoxy activated substrate through non-specific amine coupling through free amines on the lectin proteins and the epoxide groups on the glass. Arrays were neutralized and performed as previously described^26^, with the exception that Bodipy 558/568 succinimidyl ester (Thermo Scientific) was used in place of CFDA-SE. Briefly, cells were collected and washed in 1xPBS prior to being labelled for 30 minutes at 37 °C with Bodipy 558/568 succinimidyl ester. Cells were washed three times with PBS and resuspended in PBS containing 1 mM MgCl_2_ and 1 mM CaCl_2_ at 10^7^ cells/mL and 300 µL was applied to the array in a 125 µL frame without a coverslip. Cells were allowed to interact with the lectins on the array for 30 minutes and then the slides were washed three times with PBS, fixed in 4% formaldehyde and dried by centrifugation. Slides were scanned on an Innopsys InnoScan 1100AL to acquire the data of which lectins bound to the cells and analysed using Innopsys Mapix data acquisition and analysis software and Microsoft Excel for statistical analysis (Student’s unpaired *t*-test of fluorescence of background spots vs fluorescence of lectin printed spots).

## Electronic supplementary material

Supplementary Dataset S1

Supplementary Dataset S2
